# Improvements in cholesterol efflux capacity of HDL and adiponectin contribute to mitigation in cardiovascular disease risk after bariatric surgery in a cohort with morbid obesity

**DOI:** 10.1186/s13098-021-00662-3

**Published:** 2021-04-17

**Authors:** Himani Thakkar, Vinnyfred Vincent, Sakshi Sukhla, Manraj Sra, Uma Kanga, Sandeep Aggarwal, Archna Singh

**Affiliations:** 1 Department of Biochemistry, All India Institute of Medical Sciences, Room No. 3044, New Delhi, 110029 India; 2 All India Institute of Medical Sciences, New Delhi, India; 3 Department of Transplant Immunology and Immunogenetics, All India Institute of Medical Sciences, New Delhi, India; 4 Department of Surgical Disciplines, All India Institute of Medical Sciences, New Delhi, India

**Keywords:** Bariatric surgery, Cardiovascular risk, Dyslipidemia, Insulin resistance, Cholesterol efflux capacity, Adiponectin

## Abstract

**Background:**

Bariatric surgery can alleviate cardiovascular risk via effects on cardiovascular disease (CVD) risk factors such as diabetes mellitus, hypertension, and dyslipidemia. Our study aimed to assess the cholesterol efflux capacity (CEC) of HDL as a negative risk factor for CVD in individuals with obesity and identify the factors associated with improvement in CEC 3 months following bariatric surgery.

**Methods:**

We recruited 40 control individuals (mean BMI of 22.2 kg/m^2^) and 56 obese individuals (mean BMI of 45.9 kg/m^2^). The biochemical parameters, inflammatory status and CEC of HDL was measured for the obese individuals before bariatric surgery and at 3 months after surgery. The CEC was measured using a cell-based cholesterol efflux system of BODIPY-cholesterol-labelled THP-1 macrophages.

**Results:**

A significant reduction in BMI (− 17%, p < 0.001), resolution of insulin sensitivity (HOMA2-IR = − 23.4%, p = 0.002; Adipo IR = − 16%, p = 0.009) and inflammation [log resistin = − 6%, p = 0.07] were observed 3 months post-surgery. CEC significantly improved 3 months after surgery [Pre: 0.91 ± 0.13; Post: 1.02 ± 0.16; p = 0.001] despite a decrease in HDL-C levels. The change in CEC correlated with the change in apo A-I (r = 0.39, p = 0.02) and adiponectin levels (r = 0.35, p = 0.03).

**Conclusion:**

The results suggest that improvements in CEC, through improvement in adipose tissue health in terms of adipokine secretion and insulin sensitivity could be an important pathway in modulating obesity-related CVD risk.

**Supplementary Information:**

The online version contains supplementary material available at 10.1186/s13098-021-00662-3.

## Introduction

Cardiovascular disorders (CVD) account for about one-third of the global deaths. One of the modifiable risk factors for the development of CVD is obesity [1]. According to the World Health Organization, about 13% of the world’s adult population in 2016 was plagued by obesity. The obesity-induced metabolic alterations predispose an individual to multiple complications like insulin resistance, inflammation, and impaired secretion of adipokines like adiponectin [2–5]. These metabolic dysfunctions are known to promote the development of CVD [6].

Bariatric surgery (metabolic surgery) is the preferred therapeutic intervention for weight loss among individuals with severe obesity. Previous studies have reported that bariatric surgery improves obesity-associated metabolic disorders like insulin resistance and dyslipidemia [7, 8]. Emerging data show that high density lipoprotein cholesterol levels, an established negative risk factor for cardiovascular disease improves after bariatric surgery [9]. Over the past few years, studies have also demonstrated that impaired HDL functions, especially cholesterol efflux capacity (CEC) are associated with the incidence of CVD [10].

In developing countries like India, the prevalence of obesity is increasing faster than the world average and is expected to triple by 2040 [11]. Indians are also predisposed to low HDL-C levels [12]. Though bariatric surgery has been shown to ameliorate proatherogenic dyslipidemia, the present study aimed to examine the effect of bariatric surgery on the cholesterol efflux capacity of HDL in a cohort from the Indian population. We also investigated the factors associated with the change in CEC 3 months following the surgery.

## Methods

### Study design and participants

This was a longitudinal cohort study. Male and female adults aged 18 to 65 years with a BMI of 30 or more and selected for bariatric surgery were recruited from the Department of Surgical Disciplines, All India Institute of Medical Sciences, New Delhi, India. Participants were excluded if they met any of the following criteria: endocrinal causes of obesity like Cushing’s syndrome, very high risk of anesthetic complications like ASA grade IV, or severe psychiatric disorders like schizophrenia. As no estimates of CEC available from the population under study, we did an exploratory analysis and hence no sample size was calculated a priori*.*

The study included 56 individuals with obesity, of whom 30 individuals underwent Roux-en-Y gastric bypass surgery (RYGB) and twenty-six underwent sleeve gastrectomy (SG). The individuals were followed up 3 months after the surgery. In the control group, individuals with BMI < 30 kg/m^2^ and no clinical history of inflammatory diseases or autoimmune disease were included in the study.

The study was conducted after taking approval from the Institute Ethics Committee of All India Institute of Medical Sciences, New Delhi (Reference number: IEC-298/02.06.2017), and participants were enrolled after obtaining a written signed informed consent. The research was performed in accordance with relevant guidelines and regulations. All the participants were examined, and their anthropometric measurements and other medical history were recorded in a standard case record form at baseline and their 3 months postoperative visit to the bariatric surgery follow-up clinic.

### Sample collection and clinical measurements

Fasting blood samples were collected before surgery and 3 months post-surgery. Blood samples were centrifuged, and multiple aliquots of serum/plasma samples were made and stored at − 80 °C until further analysis. Biochemical parameters including glucose and lipid profile were measured on an Erba Mannheim Chem 7 semiautomated analyzer using Erba kits (Mannheim, Germany) according to the protocol described in the kit manual. Insulin measurements were made using chemiluminescence-based immunoassay using a LIAISON insulin kit on Diasorin autoanalyzer (Saluggia, Italy). Non esterified fatty acids (NEFA) levels in serum were estimated using a Randox NEFA estimation kit (Randox Laboratories, UK) according to the manual provided by the manufacturers. Apolipoprotein A-I and apolipoprotein B were determined by an immunoturbidimetric method using Randox kit with Beckmann autoanalyzer (Beckman Coulter, Brea, California, United States). Adiponectin, resistin, MCP-1 TNF- alpha, and IL-10 were measured using multiplex immunoassays (Thermo Fischer Scientific, Massachusetts, United States) on a Luminex 100/200 system.

Homeostatic model assessment method was used to assess insulin resistance (HOMA-IR) using fasting glucose and insulin according to the updated computer based HOMA2 calculator [13]. Adipose tissue insulin resistance (Adipo-IR) was calculated by multiplying free fatty acid concentration (mmol/L) by the fasting insulin concentration (pmol/L).

### Cholesterol efflux capacity (CEC) of HDL

The ability of HDL to efflux cholesterol from the macrophages was measured based on the movement of BODIPY labeled cholesterol from differentiated THP-1 cells to an acceptor that was apo B depleted serum.

The assay was performed according to a previously described protocol [10, 14, 15]. The serum samples were treated with 20% polyethylene glycol as the precipitating reagent to isolate apo B depleted serum. THP-1 human monocytes were differentiated into macrophages by treating the cells with phorbol myristate acetate (PMA) in RPMI 1640 media supplemented with 10% FBS for 48 h followed by incubation in PMA free media for 24 h.

The BODIPY-cholesterol containing labelling medium was prepared by mixing the unlabeled cholesterol (2 mM in ethanol) and BODIPY-cholesterol (5 mM in DMSO) in MEM with HEPES containing methyl-beta-cyclodextrin. The final concentration of unlabelled cholesterol and BODIPY-cholesterol were 0.1 mM and 0.025 mM respectively in the labeling medium.

Differentiated THP-1 cells were labeled with BODIPY-cholesterol by incubating the cells in labelling media for one hour. To examine the cholesterol efflux capacity of HDL, cells were incubated with 2.8% apo B depleted serum for 4 h. A concentration of 2.8% apo B depleted serum was chosen for CEC assay as it lies within the linear range and percent cholesterol efflux tends to plateau above 2.8% apo B depleted serum. Thereafter, media was removed, centrifuged and fluorescence intensity was measured (excitation 482 nm, emission 515 nm). Cells were lysed using 1% sodium cholate solution to measure the total fluorescence intensity of cells before treatment with apo B depleted serum. The percent cholesterol efflux capacity of apo B depleted serum was calculated by subtracting the fluorescence intensity value of the blank (medium incubated without apo B depleted serum) from each medium value divided by the fluorescence intensity of the cell lysates. A pooled serum from five healthy controls was included in every assay to estimate the inter-assay variability. All the samples were assayed in triplicate and the normalized cholesterol efflux capacity of the samples was calculated after normalizing to the pooled control sample CEC. The inter-assay coefficient of variation was 11%.

### Statistical analysis

Data were analyzed using STAT/IC 16.1(StataCorp LLC, Texas, US) software, and figures were created using R Core Team (2018, R: A language and environment for statistical computing; R Foundation for Statistical Computing, Vienna, Austria; URL https://www.R-project.org/) software. Data were examined for normality; normally distributed variables are presented as mean ± standard deviation and non-normally distributed as median (interquartile range). Paired t-test and Wilcoxon signed-rank test were used to compare the change in the continuous variables at baseline and 3 months after surgery. Chi-square analysis was done to compare the categorical variables. Since the reference range for HDL-C and apo A-I differs for male and female, we have presented gender specific HDL-C and apo A-I values for control and individuals with obesity before and after surgery in the study. The comparison for fraction change in variables between the two types of surgery was examined using a t-test. Pearson correlation testing was used to assess the correlation between continuous variables. Univariate linear regression analysis was performed to identify the variables associated with the change in cholesterol efflux capacity. Multivariable linear regression analysis was performed to predict the level of effect the independent variables have on the outcome variable (cholesterol efflux capacity). Multivariable linear regression analysis was done by including the variables that were found to be associated (p < 0.2) with cholesterol efflux capacity during univariate analysis. P values of < 0.05 were considered significant.

## Results

### Clinical characteristics

We analyzed samples from 40 individuals with a mean BMI of 22.2 kg/m^2^ (control group) and 56 individuals with a mean BMI of 45.9 kg/m^2^ (obese group). Characteristics of the participants are presented in Table 1. The age distribution was similar between the two groups. Individuals who were obese had significantly lower HDL-C levels (p = 0.04) and apolipoprotein A-I levels (p = 0.001) than normal weight controls. The individuals who were obese exhibited significantly lower HDL cholesterol efflux capacity (CEC) than the control group (p = 0.0002) and had lower adiponectin levels (p = 0.001).

**Table 1:** Baseline characteristics of participants

	Control (n = 40)	Obese (n = 56)	p-value
Age (years)	41.9 ± 9.7	42.9 ± 9.8	0.79
Male/female	23/17	15/41	0.001
BMI (kg/m^2^)	22.2 ± 2.0	46.1 ± 7.3	< 0.001
Diabetic, n (%)	4 (10)	22 (39.2)	0.025
Hypertensive, n (%)	4 (10)	21 (37.5)	0.04
Apo A-I (mg/dl)	150.0 ± 46.8	108.1 ± 21.5	< 0.001
Male	136.8 ± 31.3	111.4 ± 19.3	0.01
Female	168.0 ± 58.4	105.8 ± 22.9	< 0.001
Apo B (mg/dl)	75.7 ± 25.1	82.4 ± 18.6	0.1
TC (mg/dl)	163.9 ± 39.7	171.7 ± 50.7	0.37
HDL-C (mg/dl)	46.4 ± 11.0	42.7 ± 9.8	0.04
Male	43.6 ± 8.6	39.7 ± 7.2	0.16
Female	50.3 ± 13.0	43.1 ± 10.4	0.03
LDL-C (mg/dl)	102.2 ± 29.1	99.8 ± 24.5	0.84
TG (mg/dl)	105.5 ± 30.0	141.2 ± 44.0	< 0.001
VLDL-C (mg/dl)	16.1 ± 5.8	25.0 ± 9.5	< 0.001
Glucose (mg/dl)	95.0 ± 12.1	113.9 ± 32.7	0.0001
Insulin(mIU/l)	9.9 ± 5.3	21.1 ± 16.7	< 0.001
NEFA (mmol/l)	0.57 ± 0.2	0.78 ± 0.4	0.003
HOMA2-IR	1.5 ± 0.77	3.0 ± 1.8	< 0.001
Adipo-IR	34.1 (21.8–37.3)	81.8 (47.0–141.5)	< 0.001
Adiponectin (µg/ml)	12.3 ± 4.2	5.6 ± 2.2	< 0.001
CEC (A.U.)	1.04 ± 0.15	0.92 ± 0.14	0.0002

Data expressed as total (percentage) or mean ± standard deviation or median (interquartile range)

*BMI* body mass index, *Apo A-I* apolipoprotein A-I, *Apo B* apolipoprotein B, *TC* total cholesterol, *HDL-C* high density lipoprotein-cholesterol, *LDL-C* low density lipoprotein-cholesterol, *VLDL-C* very low density lipoprotein-cholesterol, *TG* triglycerides, *NEFA* non-esterified fatty acids, *HOMA2-IR* homeostatic model assessment method-insulin resistance, *Adipo-IR* adipose tissue-insulin resistance, *CEC* cholesterol efflux capacity

Among the 56 obese individuals, 30 underwent Roux-en-Y gastric bypass surgery (RYGB), and twenty-six underwent sleeve gastrectomy (SG). Among the study cohort, 41 individuals reported for follow up as per schedule, while 15 did not visit the follow-up clinic for the 3-month follow-up. The mean age of the participants in the obese group was 42 years. 44% of individuals with obesity had diabetes, and 37% were hypertensive.

The baseline characteristics of the obese individual between surgery groups were similar for age (RYGB: 42.7 ± 9.0 vs. SG: 43.1 ± 10.8 years), BMI (RYGB: 45.2 ± 5.5 vs. SG: 47.0 ± 8.9 kg/m^2^), and other biochemical parameters except for glucose and apolipoprotein B levels. The number of obese individuals with diabetes was 17 in the RYGB surgery group and 5 in the SG group. The complete comparison of demographic and biochemical variables between the two types of surgeries is presented in Additional file 1: Table S1.

### Effect of bariatric surgery

Bariatric surgery significantly reduced BMI in obese individuals at 3 months following surgery. The BMI decreased by 17% (Pre: 45.8 ± 7.2, Post: 37.9 ± 6.3, p < 0.001), and the percentage decrease in BMI was similar in both types of surgery. We analyzed the insulin sensitivity status of obese individuals undergoing bariatric surgery before and 3 months after surgery. Fasting glucose [5% (Pre vs Post: 116.7 ± 36.6 vs 100 ± 15.8 mg/dl); p = 0.009], insulin [27.3%; Pre vs Post: 16.5 (12.3–13.2) vs 9.75 (7.1–13.2); p < 0.001] and HOMA2-IR [23.4%; Pre vs Post: 2.5 (2.5–3.8); p = 0.002] were significantly decreased post-surgery. The change in glucose levels (Mean: − 15% vs. 16%, p = 0.03) was greater in the RYGB group vs. SG, while the decrease in insulin levels was similar in both the groups, as a result of which the percent change in HOMA2-IR was significant in RYGB group compared to SG group. While there was a decrease in NEFA levels after surgery, it was not significant. The ADIPO-IR decreased significantly after surgery (16%, p = 0.009), with the magnitude of decrease more significant in the RYGB group. The comparison of variables between individuals with obesity before surgery and 3 months after surgery are represented in Table 2.

**Table 2:** Change in the variables 3 months after surgery

Variable	Before surgery (n = 41)	After surgery (n = 41)	Percent change [95% CI]	p-value
BMI (kg/m^2^)	45.8 ± 6.3	37.9 ± 7.6	− 17.4 [(− 19.5) to (− 15.3)]	< 0.001
Glucose (mg/dl)	116.7 ± 36.6	100 ± 15.8	− 5.0 [(− 20.7) to (10.6)]	0.009
Insulin (mIU/l)	16.5 (12.3–13.2)	9.75 (7.1–13.2)	− 27.3 [(− 50.6) to (− 4.0)]	< 0.001
HOMA2-IR	2.5 (2.5–3.8)	1.5 (1.2–2.2)	− 23.4 [(− 49.6) to (− 2.8)]	0.002
NEFA (mmol/l)	0.78 ± 0.34	0.74 ± 0.32	29.0 [(− 13.8) to (71.6)]	0.67
ADIPO-IR	81.2 (48.0–137.0)	57.5 (25.5–94.1)	− 16.0 [(− 58.4) to (26.3)]	0.009
Hb1Ac	7.1 ± 1.8	5.9 ± 0.94	− 15.1 [(− 19.8) to (− 10.4)]	< 0.001
Apo A-I (mg/dl)	107.1 ± 22.3	129.8 ± 20.2	25.7 [(16.0) to (34.0)]	< 0.001
Male	114.8 ± 25.3	128.7 ± 17.9	13.8 [(2.4) to (25.3)]	0.04
Female	105.2 ± 21.6	130.1 ± 21.0	27.7 [(16.8) to (38.5)]	< 0.001
Apo B (mg/dl)	81.6 ± 19.4	101.7 ± 18.2	26.0 [(16.3) to (35.1)]	< 0.001
TC (mg/dl)	176.3 ± 54.7	166 ± 29.8	− 2.2 [(− 8.3) to (4.0)]	0.18
HDL-C (mg/dl)	42.4 ± 9.9	39.2 ± 6.1	− 3.8 [(− 10.6) to (3.1)]	0.044
Male	40.5 ± 7.4	37.5 ± 7.0	− 4.7 [(− 20.8) to (11.3)]	0.40
Female	43.0 ± 10.7	39.7 ± 5.8	− 3.4 [(− 11.5) to (4.6)]	0.08
LDL-C (mg/dl)	102.9 ± 27	105.1 ± 21.3	10.2 [(− 5.6) to (26.0)]	0.67
TG (mg/dl)	141.4 ± 41.2	118.5 ± 43.4	− 12.5 [(− 23.1) to (− 1.2)]	0.003
VLDL-C (mg/dl)	25.5 ± 10	22.4 ± 9.1	0.5 [(− 18.0) to (19.0)]	0.09
CEC	0.91 ± 0.13	1.02 ± 0.16	11.5 [(6.1) to (17.0)]	0.00017
Adiponectin (µg/ml)	5.6 ± 2.2	6.8 ± 2.5	32.2 [(15.0) to (49.4)]	0.005
Log resistin	2.3 ± 0.72	2.0 ± 0.80	− 6.0 [(− 18.0) to (5.9)]	0.07
Log MCP-1	1.8 ± 0.43	1.7 ± 0.50	− 2.4 [(− 12.9) to (8.0)]	0.17
IL-10 (pg/ml)	0.25 (0.05–0.7)	0.39 (0.14–0.56)	47.8 [(68.0) to (879.2.0)]	0.64
TNF-alpha (pg/ml)	2.3 ± 1.8	2.4 ± 1.3	30.0 [(0.0) to (60.3)]	0.83

Data expressed as mean ± standard deviation or median (interquartile range) and mean percent change with 95% confidence interval

*BMI* body mass index, *HOMA2-IR* homeostatic model assessment method-insulin resistance, *NEFA* non-esterified fatty acids, *Adipo-IR* adipose tissue-insulin resistance, *HbA1c* glycated hemoglobin, *Apo A-I* apolipoprotein A-I, *Apo B* apolipoprotein B, *TC* total cholesterol, *HDL-C* high density lipoprotein-cholesterol, *LDL-C* low density lipoprotein-cholesterol, *TG* triglycerides, *VLDL-C* very low density lipoprotein-cholesterol, *CEC* cholesterol efflux capacity, *MCP-1* monocyte chemoattractant protein-1, *IL-10* interleukin 10, *TNF-alpha* tumor necrosis factor-alpha

We assessed the change in lipid profile 3 months after the bariatric surgery. Total cholesterol and VLDL-C changed minimally. HDL-C decreased by 4% (p = 0.04), with the decrease being evident in both types of surgeries but a trend towards greater reduction in participants who underwent RYGB. Despite the decrease in HDL-C levels, a significant increase in apolipoprotein A-I levels (26%, p < 0.001) was observed 3 months post-surgery. The triglyceride levels decreased significantly (12%; p = 0.003) at 3 months with SG producing greater reduction compared to RYGB. Apo B levels and LDL-C significantly increased by 26% (p < 0.001) and 10% (p = 0.67) respectively. The summary of the percent changes in two types of surgery is shown in Table 3 and Additional file 1: Table S2.

**Table 3:** Change in variables from baseline at 3 months after Roux-en-Y gastric bypass (malabsorptive) and sleeve gastrectomy (restrictive) surgery

Variable	Sleeve gastrectomy (n = 14)			Roux-en-Y gastric bypass (n = 27)		
	Before surgery	After surgery	p	Before surgery	After surgery	p
BMI (kg/m^2^)	46.9 ± 10.1	39 ± 8.2	0.0001	44.8 ± 4.5	36.9 ± 3.9	0.0001
Glucose (mg/dl)	92.3 ± 22.2	94.5 ± 16	0.8	131.1 ± 37.1	104.1 ± 14.8	0.004
Insulin (mIU/l)	15.1 (11.8–21.8)	8.3 (4.3–12.0)	0.009	19.2 (12.8–27.6)	10.1 (7.5–14.3)	0.001
HOMA2- IR	2.3 (1.6–3.0)	1.2 (0.8–1.9)	0.12	3.1 (2.1–4.3)	1.5 (1.1–2.2)	0.0003
NEFA	0.73 ± 0.34	0.78 ± 0.2	0.57	0.8 ± 0.35	0.72 ± 0.33	0.47
ADIPO-IR	118.2 ± 80.2	92 ± 81.4	0.68	107.3 ± 77.6	51.8 ± 9.1	0.005
Hb1Ac	6.2 ± 1	5.3 ± 0.94	0.007	7.7 ± 2.0	6.2 ± 0.8	0.0007
Apo A-I (mg/dl)	117.5 ± 21.3	135.5 ± 25.7	0.06	103.5 ± 23.1	127.8 ± 18.6	0.006
Male	122.3 ± 32.1	133.5 ± 14.0	0.09	106.7 ± 13.1	123.3 ± 18.5	0.01
Female	111.3 ± 14.8	137.5 ± 28.2	0.04	102.8 ± 25.0	126.8 ± 19.0	0.002
Apo B (mg/dl)	87.8 ± 20.4	103.8 ± 19.1	0.02	75.4 ± 17.0	99.2 ± 16.5	0.001
TC (mg/dl)	193.2 ± 80.9	176.8 ± 34.6	0.35	163.1 ± 22.9	160.7 ± 22.6	0.66
HDL-C (mg/dl)	43.9 ± 11.1	42.3 ± 5.2	0.53	42.1 ± 9.3	37 ± 5.9	0.017
Male	41.7 ± 8.0	41.3 ± 5.0	0.92	39.3 ± 7.3	31.5 ± 4.2	0.03
Female	45.5 ± 12.7	42.6 ± 6.0	0.44	42.6 ± 9.7	38.2 ± 5.6	0.04
LDL-C (mg/dl)	103.5 ± 26.6	116.1 ± 33.0	0.12	99.5 ± 26.2	97.5 ± 29.4	0.74
TG (mg/dl)	146.9 ± 51.1	116.5 ± 36.9	0.009	137.6 ± 35.9	122.1 ± 48.7	0.14
VLDL-C (mg/dl)	26.6 ± 11.7	21.9 ± 8.7	0.16	24.6 ± 9.3	23.1 ± 9.6	0.5
CEC (A.U.)	0.89 ± 0.1	1.03 ± 0.18	0.012	0.92 ± 0.14	1.0 ± 0.14	0.006
Adiponectin (µg/ml)	6.0 ± 1.3	7.7 ± 1.5	0.001	5.4 ± 2.4	6.7 ± 2.7	0.02
Log resistin	2.4 ± 0.7	1.9 ± 0.67	0.06	2.3 ± 0.16	2.0 ± 0.78	0.09
Log MCP-1	1.6 ± 0.4	1.6 ± 0.47	0.97	1.9 ± 0.41	1.6 ± 0.53	0.08
IL-10 (pg/ml)	0.1 (0.01–0.44)	0.2 (0.12–0.56)	0.62	0.33 (0.1–0.8)	0.2 (0.11–0.54)	0.21
TNF-alpha (pg/ml)	2 ± 0.8	3 ± 1.4	0.05	2.5 ± 2.2	2.2 ± 1.3	0.6

Data expressed as mean ± standard deviation or median (interquartile range)

*BMI* body mass index, *HOMA2-IR* homeostatic model assessment method-insulin resistance, *NEFA* non-esterified fatty acids, *Adipo-IR* adipose tissue-insulin resistance, *HbA1c* glycated hemoglobin, *Apo A-I* apolipoprotein A-I, *Apo B* apolipoprotein B, *TC* total cholesterol, *HDL-C* high density lipoprotein-cholesterol, *LDL-C* low density lipoprotein-cholesterol, *VLDL-C* very low density lipoprotein-cholesterol, *TG* triglycerides, *CEC* cholesterol efflux capacity, *MCP-1* monocyte chemoattractant protein-1, *IL-10* interleukin 10, *TNF-alpha* tumor necrosis factor-alpha

We evaluated the levels of adipokines in serum such as adiponectin, resistin and MCP-1. The adiponectin levels increased significantly (33%, p = 0.005), while there was a non-significant decrease in resistin (− 6%, p = 0.07) and MCP-1 (− 2.4%, p = 0.17) levels postoperatively.

### HDL function

**Fig. 1:**
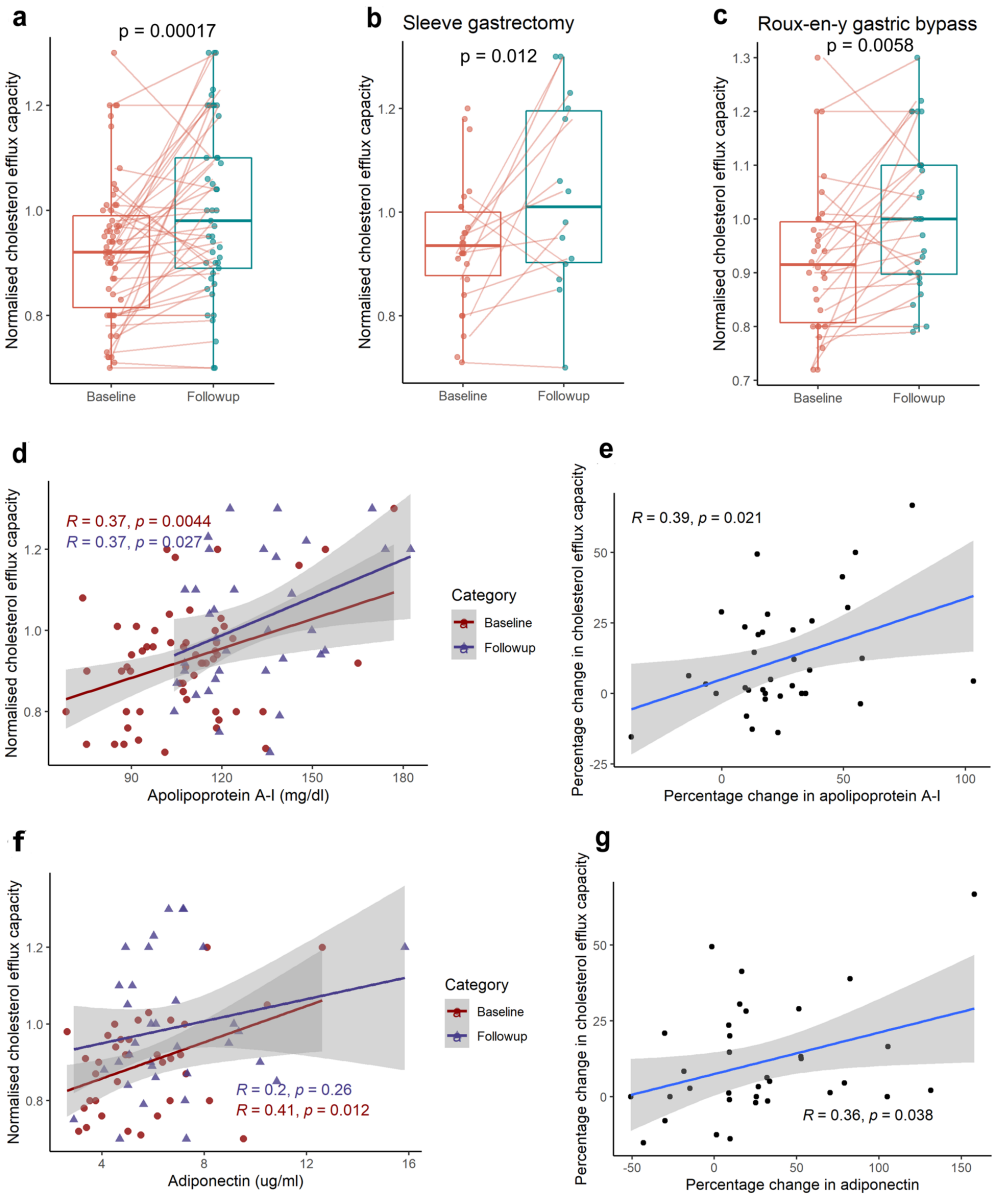
Change in Cholesterol efflux capacity (CEC) of HDL and its correlation with variables. **a** Change in CEC of HDL in individuals with obesity at baseline and 3 months post bariatric surgery (Follow-up). **b**, **c** Change in variables from baseline at 3 months after sleeve gastrectomy and Roux-en-Y gastric bypass surgery, respectively. Lines connecting the dots indicate the change in individual subject at 3 months. Change in CEC after surgery was analysed using paired t test. **d**, **f** Correlation of CEC with apolipoprotein A-I and adiponectin, respectively at baseline and 3 months after surgery. **e**, **g** Correlation of change in CEC with change in apolipoprotein A-I and adiponectin. R represents Pearson correlation coefficient and line represents the regression line

The HDL functional capacity to accept cholesterol from the macrophages was analyzed both pre-and post-surgery using THP-1 monocytic cells. We measured the capacity of apo B depleted serum (containing HDL as cholesterol acceptor) to accept cholesterol from the macrophages loaded with fluorescently labeled cholesterol. We observed that there was a significant improvement in the CEC of HDL (Pre: 0.91 ± 0.13; Post: 1.0 ± 0.16; p = 0.001) 3 months post-surgery (Fig. 1a). The mean percent change in cholesterol efflux capacity was 11%, and the percent change was similar between the surgeries (Fig. 1b, c).

We also analyzed the difference in the biochemical parameters between individuals with obesity and diabetes and individuals with obesity and without diabetes. At baseline, all biochemical parameters except glucose and Hb1Ac levels were similar between groups with and without diabetes (Additional file 1: Table S3). Non-diabetic individuals had lower MCP-1 and TNF-alpha levels than diabetics, and the MCP-1 levels were also significantly lower in non-diabetics than people with diabetes after surgery. The percent change in the biochemical parameters was similar in both diabetic and non-diabetic individuals. The baseline CEC was higher in individuals who were non-diabetic than in diabetics (Diabetic: 0.89 ± 0.16 vs. Nondiabetic: 0.94 ± 0.13) (Additional file 1: Table S4). The change in HDL's cholesterol efflux capacity after bariatric surgery was similar in individuals with and without diabetes. (Additional file 1: Table S5).

We performed a regression analysis between clinical variables and CEC at baseline and post-surgery to identify the variables associated with CEC variation. The baseline CEC values were significantly correlated with pre apolipoprotein A-I levels (r = 0.37, p = 0.004) (Fig. 1d) and were negatively correlated with pre total cholesterol levels (r = − 0.29, p = 0.02). CEC was also strongly correlated with adiponectin levels at baseline (r = 0.41, p = 0.01) (Fig. 1f). The association of apo A-I levels and CEC remained significant even after surgery, while a positive trend was observed with adiponectin but was not significant (Table 4, Fig. 1f). The percentage change in CEC was also significantly correlated with the percentage change in apo A-I (r = 0.39, p = 0.021) and adiponectin levels (r = 0.36, p = 0.038) (Fig. 1e, g).

**Table 4:** Correlation coefficients (r) for cholesterol efflux capacity with variables

Variable	Correlation of CEC with variables at baseline		Correlation of CEC with variables at 3 months		Correlation of percent change in CEC with percent change in variables	
	r	p-value	r	p-value	r	p-value
BMI (kg/m^2^)	− 0.09	0.50	− 0.04	0.79	0.12	0.50
HOMA2-IR	− 0.12	0.38	0.22	0.27	0.32	0.21
Apo A-I (mg/dl)	0.37	**0.004**	0.37	**0.02**	0.39	**0.02**
Apo B (mg/dl)	− 0.02	0.89	0.07	0.68	0.001	1.00
TC (mg/dl)	− 0.29	**0.02**	0.02	0.92	− 0.004	0.97
HDL-C (mg/dl)	0.17	0.19	− 0.06	0.73	0.08	0.65
LDL-C(mg/dl)	− 0.29	**0.02**	− 0.23	0.17	− 0.11	0.52
TG (mg/dl)	− 0.05	0.71	0.33	0.05	0.13	0.47
Adiponectin (µg/ml)	0.41	**0.01**	0.20	0.25	0.35	**0.03**
Log resistin	− 0.18	0.26	− 0.06	0.72	0.09	0.60
TNF alpha (pg/ml)	0.17	0.36	0.14	0.5	0.17	0.55
IL-10 (pg/ml)	− 0.08	0.62	0.26	0.13	− 0.14	0.43

*BMI* body mass index, *HOMA2-IR* homeostatic model assessment method-insulin resistance, *Apo A-I* apolipoprotein A-I, *Apo B* apolipoprotein B, *HDL-C* high density lipoprotein-cholesterol, *LDL-C* low density lipoprotein-cholesterol, *TG* triglycerides, *CEC* cholesterol efflux capacity, *IL-10* interleukin 10, *TNF-alpha* Tumor necrosis factor-alpha. r represents Pearson correlation coefficient. Bold emphasis denotes a significant p-value i.e. < 0.05

The regression analysis was performed using changes in biochemical variables with change in CEC. It showed that changes in CEC were most strongly associated with the changes in apoA-I and adiponectin levels (Table 5).

**Table 5:** Multivariable linear regression model of change in variables with change in cholesterol efflux capacity 3 months after surgery

Variables	Univariate analysis			Multivariable analysis		
	β	SE	p-value	β	SE	p-value
BMI (kg/m^2^)	0.38	0.57	0.50			
Glucose (mg/dl)	− 0.07	0.13	0.60			
Apo A-1 (mg/dl)	0.3	0.11	**0.02**	0.2	0.14	0.24
HDL-C (mg/dl)	0.07	0.15	0.65			
LDL-C (mg/dl)	− 0.05	0.08	0.52			
TG (mg/dl)	0.06	0.08	0.50			
TC (mg/dl)	− 0.003	0.15	1.00			
Adiponectin (µg/ml)	0.13	0.06	**0.03**	0.11	0.08	0.18
Log resistin	0.05	0.09	0.60			
IL-10 (mg/dl)	− 0.002	0.002	0.43			
TNF-alpha (mg/dl)	0.03	0.05	0.55			

*SE* standard error, *BMI* body mass index, *Apo A-I* apolipoprotein A-I, *Apo B* apolipoprotein B, *HDL-C* high density lipoprotein-cholesterol, *LDL-C* low density lipoprotein-cholesterol, *TG* triglycerides, *CEC* Cholesterol efflux capacity, *IL-10* interleukin 10, *TNF-alpha* tumor necrosis factor-alpha. Bold emphasis denotes a significant p-value i.e. < 0.05

## Discussion

The work presented in this manuscript is the first study to demonstrate the effect of bariatric surgery on HDL functionality in terms of CEC in the Asian Indian population. It is particularly pertinent given the higher propensity of metabolic syndrome and the distinct adipose tissue distribution and biological dynamics reported in the population from the Indian subcontinent compared to those of Caucasian ethnicity [16–18]. We also demonstrated for the first time that the improvement in CEC after bariatric surgery is evident as early as 3 months postoperatively. We observed that despite some decrease in HDL-C levels, there was a significant improvement in CEC 3 months post-surgery irrespective of the type of surgery (SG and RYGB). The CEC improvement at 3 months after surgery was primarily explained by changes in apo A-I and adiponectin levels.

HDL's capacity to promote cholesterol efflux from macrophages is known to have a strong negative association with the incidence of CVD independent of HDL-C levels [19], and we have previously demonstrated that low CEC is associated with higher odds of having acute coronary syndrome [14]. We observed a lower CEC in individuals with obesity than control subjects in concordance with other published studies [20]. The individuals with obesity in our study also had significantly lower levels of apo A-1 and adiponectin in circulation along with atherogenic dyslipidemia (high TG and low HDL-C) and insulin resistance (high HOMA-IR and Adipo-IR). All these are established risk factors for the development of CVD in obesity.

We observed that bariatric surgery significantly improved CEC 3 months after surgery. As Indians are highly predisposed to metabolic alterations associated with obesity and have a high prevalence of CVD [21], we focussed on identifying the factors associated with CEC improvement in obese individuals after bariatric surgery. After bariatric surgery, improvement in CEC was most significantly associated with an increase in apo A-1 levels in circulation. Apo A-1 is the major protein component of HDL, and the levels of apo A-1 have been shown to modulate the CEC of HDL. Increase in apo A-I levels could explain improvement in CEC despite a reduction in HDL-C levels. As adipose tissue mass reduction is the most significant outcome of bariatric surgery, we further evaluated adipose tissue-related factors that could affect CEC and apo A-1 levels.

Adipose tissue is a metabolically active endocrine organ that releases many cytokines and bioactive mediators known as adipokines. These include adiponectin, resistin, interleukin-6 (IL-6), tumor necrosis factor-α (TNF-α), and monocytic chemotactic protein (MCP-1) that influence not only body weight homeostasis but also insulin resistance, diabetes, lipid levels, inflammation, and atherosclerosis development [22]. In this study, we observed a significant increase in adiponectin after 3 months of bariatric surgery. Adiponectin is an adipokine that is known to be inversely associated with the incidence of obesity-linked CVD. Adiponectin exerts protective effects against atherosclerosis due to its anti-inflammatory and antiatherogenic properties and suppresses foam cell formation [23, 24]. Adiponectin has been shown to be positively associated with plasma HDL-C levels and increased the expression and secretion of apo A-I in the liver through PPAR-α and LXR signaling [25]. Thus, the increase in adiponectin levels could partly explain the increase in apo A-I levels after surgery and hence the improvement in CEC. In addition to increasing apo A-I levels, adiponectin also increases the expression of transporters ABCA1 and ABCG1 [26] involved in the assembly of HDL particles and reverse cholesterol transport. Our previous work has reported lower ABCA1 expression levels in visceral adipose tissue of individuals with obesity than controls [27]. Therefore, the upregulation of transporters like ABCA1 in peripheral tissues and apo A-1 by adiponectin accelerates HDL biogenesis and could also explain the improvement in CEC after bariatric surgery [28].

It is known that bariatric surgery induces alterations in gastrointestinal anatomy and physiology that modulates the release of gastrointestinal peptides like glucagon-like peptide-1 (GLP-1). Previous studies have demonstrated that GLP-1 receptor agonists induce adiponectin secretion in adipocytes both in vitro and in vivo through Sirt1/Foxo-1 signaling and decrease inflammatory expression adipokines like MCP-1 and IL-6 [29, 30]. In agreement with these studies, we observed a significant increase in adiponectin levels and a substantial reduction in resistin and MCP-1 levels 3 months post-surgery, indicating an improvement in adipose tissue health.

Insulin resistance is one of the critical factors involved in the pathogenesis of obesity-induced CVD [6]. In the setting of diabetes, altered HDL subfraction distribution in terms of an increased number of small dense Apo A-I depleted HDL particles have been reported to be the cause for impaired HDL functions [31–33]. A lower CEC was observed in individuals with obesity and diabetes compared to obese individuals without diabetes. Studies have demonstrated that hyperglycemia induces alteration in HDL metabolism and function [34, 35]. Consistent with prior findings in the literature [9, 36, 37], we observed an improvement in insulin sensitivity 3 months following surgery. Improvement in insulin sensitivity could also be one of the contributing factors to a decrease in CVD risk after bariatric surgery.

Obesity is known to be a chronic low-grade inflammatory state which promotes the progression of atherosclerosis. Resistin, an inflammatory cytokine secreted by the adipose tissue, is suggested to have a role in the development of insulin resistance. It also induces the secretion of other inflammatory cytokines like IL-6 and TNF-alpha [38, 39]. We observed a substantial decrease in levels of resistin after bariatric surgery in obese individuals. Numerous studies state that high inflammatory conditions lead to remodeling of HDL proteins and impairment in HDL functions [40]. Data from the current study raise the possibility that chronic inflammatory conditions could cause low HDL CEC in obesity, which improved after bariatric surgery.

Previous studies exploring the effect of bariatric surgery on cholesterol efflux capacity of HDL have reported some conflicting findings. We did not observe any difference between the two types of bariatric surgery (SG and RYGB) in CEC improvement. This observation contrasts with a previously published study where authors demonstrated that the change in CEC after bariatric surgery was dependent on the type of surgery [41]. The two studies' key differences were the duration of follow up (6 months) and the cell line used to quantify CEC (a mouse macrophage cell line J774A.1). The only factor that showed a significant difference between the two types of surgery in our study was improved plasma glucose. The reduction in glucose levels was greater in the RYGB group compared to the SG group. This outcome could partially be due to the higher percentage of individuals with diabetes in the RYGB group.

A study by Kjellmo et al. observed no significant change in CEC after bariatric surgery, although similar methodology was used to assess the CEC [42]. The difference in study populations in terms of ethnicity could be the reason for inconsistent findings. Another study by Lorkowski et al., examined the effect of bariatric surgery (1 and 5 year post-operatively) on HDL functions in individuals with obesity and diabetes. In this study group, authors reported an improvement in CEC only at 5 years after the surgery, while our study observed a significant increase in CEC (both overall and in subset of obese individuals with diabetes) at 3 months after surgery [43]. Further, in contrast to the above study which used radiolabelled cholesterol and mouse macrophage system, we have used fluorescently labelled cholesterol and human monocyte cell line to measure CEC in the serum of study participants. Thus, the differences in composition of the study group analysed, the assay methodological details and follow-up duration make it difficult for a strict comparison to be made between the findings from the two studies, although overall, both the studies observed improvement in CEC independent of the surgery type.

Our study has some limitations. A limited sample size is one of them. In the study, an improvement in apo A-I levels was observed despite a decrease in HDL-C levels. This suggests that HDL subfraction distribution could have been altered, but we did not analyze this apsect. Since HDL is a heterogenous particle and HDL subfractions differ in composition and function, exploring the distribution and composition of HDL particles would be relevant in the context of bariatric surgery. Larger sample size and more extended follow-up periods are also needed to completely understand HDL’s biology in the context of bariatric surgery and CVD risk. While the sample size is small, we believe that given the unique nature of the study group in terms of underlying metabolic complexity, our study makes a valuable contribution to the limited data available on the analysed variables. We would also like to state the sub-group analysis by surgery type was done in a very small group and would require further validation in a larger cohort. Another limitation is that we could not identify the underlying mechanism for increased apo B and LDL-C levels observed 3 months after bariatric surgery. Unlike mouse macrophage cell line that has inducible ABCA1, the differentiated THP-1 cells have constitutive ABCA1 expression because of which it was not possible to differentiate between ABCA1-dependent and ABCA1-independent cholesterol efflux of HDL using THP-1 system in this study.

## Conclusion

In conclusion, the data demonstrate that improvement in apo A-I and adiponectin levels are the critical predictors for CEC improvement in obese individuals after bariatric surgery. The variation in adiponectin production may be a crucial modulator of apo A-I synthesis and hence the HDL functionality, representing a mechanism for decreasing cardiovascular risk associated with obesity. While there are other determinants and markers of CVD risk in individuals with obesity, our work highlights improvements in CEC and associated adipose driven changes as one of the CVD risk mitigating effects of bariatric surgery. The current study results thus add evidence in support of the evolving concept that anti-inflammatory factors and adipokines may play a central role in modulating obesity-related CVD risk.

## Supplementary Information

**Additional file 1: Table S1.** Baseline characteristics of obese participants. **Table S2.** Comparison of percent change in variables between surgeries. **Table S3.** Comparison of the baseline characteristics of individuals with obesity and diabetes and individuals with obesity without diabetes. **Table S4.** Comparison of the variables after surgery in individuals with obesity and diabetes and individuals with obesity without diabetes. **Table S5.** Comparison of the percent change in variables after surgery between individuals with obesity and diabetes and individuals with obesity and non-diabetic

## Data Availability

Not applicable.

## Abbreviations

ADIPO-IR: Adipose tissue- insulin resistance
Apo A-I: Apolipoprotein A-I
Apo B: Apolipoprotein B
BMI: Body Mass Index
CEC: Cholesterol efflux capacity
CVD: Cardiovascular disease
HDL-C: High density lipoprotein-cholesterol
HOMA2-IR: Homeostatic model assessment model-insulin resistance
LDL-C: Low density lipoprotein-cholesterol
MCP-1: Monocytic chemotactic protein
RYGB: Roux-en-Y gastric bypass surgery
SG: Sleeve gastrectomy
TC: Total Cholesterol
TG: Triglyceride
TNF-alpha: Tumor necrosis factor-alpha
VLDL: Very low density lipoprotein-Cholesterol
