# Correction to: Improvements in cholesterol efux capacity of HDL and adiponectin contribute to mitigation in cardiovascular disease risk after bariatric surgery in a cohort with morbid obesity

**DOI:** 10.1186/s13098-021-00684-x

**Published:** 2021-06-14

**Authors:** Himani Thakkar, Vinnyfred Vincent, Sakshi Shukla, Manraj Sra, Uma Kanga, Sandeep Aggarwal, Archna Singh

**Affiliations:** 1 Department of Biochemistry, All India Institute of Medical Sciences, Room No. 3044, New Delhi, 110029 India; 2 All India Institute of Medical Sciences, New Delhi, India; 3 Department of Transplant Immunology and Immunogenetics, All India Institute of Medical Sciences, New Delhi, India; 4 Department of Surgical Disciplines, All India Institute of Medical Sciences, New Delhi, India

## Correction to: Diabetol Metab Syndr (2021) 13:46 https://doi.org/10.1186/s13098-021-00662-3

It was highlighted that the original article [1] contained an error in the family name of author Sakshi Shukla. It was incorrectly captured as Sakshi Sukhla. The original article has been updated.
