# Supplementary material for: Improvements in cholesterol efflux capacity of HDL and adiponectin contribute to mitigation in cardiovascular disease risk after bariatric surgery in a cohort with morbid obesity
Source: Diabetol Metab Syndr. 2021 Apr 17;13:46. doi: 10.1186/s13098-021-00662-3 (PMC8053301; doi:10.1186/s13098-021-00662-3)
Supplement: Supplementary file 1 — Additional file 1: Table S1. Baseline characteristics of obese participants. Table S2. Comparison of percent change in variables between surgeries. Table S3. Comparison of the baseline characteristics of individuals with obesity and diabetes and individuals with obesity without diabetes. Table S4. Comparison of the variables after surgery in individuals with obesity and diabetes and individuals with obesity without diabetes. Table S5. Comparison of the percent change in variables after surgery between individuals with obesity and diabetes and individuals with obesity and non-diabetic [file 13098_2021_662_MOESM1_ESM.docx]

**Table S1: Baseline characteristics of obese participants**

|  | **All subjects (n = 56)** | **Sleeve gastrectomy (n**  **= 26)** | **Roux-en-Y gastric bypass (n =30)** |
| --- | --- | --- | --- |
| Age (years) | 42.9 ± 9.8 | 43.1 ± 10.8 | 42.7 ± 9.0 |
| Diabetic, n (%) | 22 (39.2) | 5 (19.2) | 17 (56.7) |
| BMI (kg/m2) | 46.1 ± 7.3 | 47.0 ± 8.9 | 45.2 ± 5.5 |
| Glucose (mg/dl) | 113.9 ± 32.7 | 97.7 ± 21.9 | 126.8 ± 34.4* |
| Insulin (mIU/l) | 16.8 (12.3 – 24.0) | 15.1 (11.8 – 21.8) | 19.2 (12.8 – 27.6) |
| HOMA2-IR | 2.5 (2.0 – 3.8) | 2.3 (2.1 – 4.4) | 3.1 (1.6 – 3.0) |
| NEFA (mmol/l) | 0.78 ± 0.4 | 0.75 ± 0.44 | 0.81 ± 0.37 |
| ADIPO-IR | 81.2 (48.0 – 137.0) | 59.4 (42.3 – 111.9) | 82.6 (53.7 – 137.0) |
| Hb1Ac | 6.7 ± 1.7 | 6.0 ± 0.85 | 7.2 ± 2.0 |
| Apo A-I (mg/dl) | 108.1 ± 21.5 | 113.2 ± 21.3 | 103.8 ± 21.1 |
| Apo B (mg/dl) | 82.4 ± 18.6 | 89.3 ± 18.6 | 76.4 ± 16.6** |
| TC (mg/dl) | 171.7 ± 50.7 | 182.9 ± 69.6 | 162.0 ± 22.1 |
| HDL-C (mg/dl) | 42.7 ± 9.8 | 42.8 ± 10.3 | 42.6 ± 9.5 |
| LDL-C (mg/dl) | 99.8 ± 24.5 | 101.5 ± 25.2 | 98.3 ± 25.0 |
| TG (mg/dl) | 141.2 ± 44.0 | 148.7 ± 51.9 | 134.5 ± 34.9 |
| VLDL-C (mg/dl) | 25.0 ± 9.5 | 25.7 ± 10.5 | 24.4 ± 8.7 |

Data expressed as percentage or mean ± standard deviation or median (interquartile range). BMI, Body mass index; HOMA2-IR, Homeostatic model assessment method-insulin resistance; NEFA, Non- esterified fatty acids; Adipo-IR, adipose tissue-insulin resistance; Hb1Ac; glycated hemoglobin; Apo A-I, Apolipoprotein A-I; Apo B, Apolipoprotein B; TC, Total cholesterol; HDL-C, High density lipoprotein- cholesterol; LDL-C, Low density lipoprotein-cholesterol; TG, Triglycerides; VLDL-C, Very low density lipoprotein-cholesterol. *p<0.05, **p<0.01

**Table S2: Comparison of percent change in variables between surgeries**

| **Variable** | **Sleeve gastrectomy (n =14)** | **Roux-en-Y gastric bypass (n**  **=27)** |  |
| --- | --- | --- | --- |
|  | **Mean percent change [95% CI]** | **Mean percent change [95% CI]** | **p-value** |
| BMI (kg/m2) | -16.3 [(-20.0) - (-12.7)] | -18.4 [(-21.0) - (-15.8)] | 0.60 |
| Glucose (mg/dl) | 16 [(-27.6) – (59.0)] | -15.4 [(-26.4) - (-4.4)] | 0.03 |
| Insulin (mIU/l) | -17.6 [(-72.0) – (36.6)] | -32.4 [(-54.0) – (-10.8)] | 0.55 |
| HOMA2-IR | -4.6 [(-78.0) – (68.9)] | -30.8 [(-55.7) – (-5.8)] | 0.23 |
| NEFA (mmol/l) | 23.5 [(-20.0) – (67.0)] | 17.0 [(-26.4) – (60.5)] | 0.85 |
| ADIPO-IR | 5.1 [(-82.7) – (93.0)] | -18.5 [(-76.8) – (40.0)] | 0.65 |
| Hb1Ac | -13.4 [(-22.1) – (-5.5)] | -16.2 [(-23.1) – (-9.4)] | 0.58 |
| Apo A-I (mg/dl) | 17.0 [(1.2) – (32.7)] | 28.2 [(15.2) – (41.1)] | 0.27 |
| Apo B (mg/dl) | 21.7 [(1.3) – (42.1)] | 30.4 [(18.2) – (42.6)] | 0.30 |
| TC (mg/dl) | -2 [(-13.6) – (8.7)] | 00 [(-7.4) – (7.5)] | 0.69 |
| HDL-C (mg/dl) | 0.5 [(-10.4) – (11.5)] | -8.7 [(-18.2) – (1.0)] | 0.19 |
| LDL-C (mg/dl) | 21.9 [(-9.0) – (53.3)] | 4.8 [(-13.7) – (23.4)] | 0.30 |
| TG (mg/dl) | -17.0 [(-29.0) – (-5.1)] | -7.8 [(-26.4) – (10.8)] | 0.44 |
| VLDL-C (mg/dl) | 1.1 [(-36.7) – (39.0)] | 2.6 [(-19.0) – (24.2)] | 0.94 |
| CEC (A.U.) | 17 [(5.0) – (29.0)] | 10.1 [(3.4) – (16.7)] | 0.48 |
| Adiponectin (µg/ml) | 32.8 [(11.5) – (54.0)] | 38.1 [(12.5) – (63.8)] | 0.78 |
| Log Resistin | -17.1 [(-34.7) – (0.5)] | -7.1 [(-20.6) – (6.4)] | 0.36 |
| Log MCP-1 | 7.0 [(-15.5) – (29.2)] | -7.9 [(-21.2) – (5.6)] | 0.22 |
| IL-10 (pg/ml) | 906.5 [(-285.3) – (2090.3)] | 324.3 [(-85.1) – (733.0)] | 0.21 |
| TNF-alpha (pg/ml) | 57.7 [(-4.6) – (120.0)] | 19.2 [(-22.4) – (60.7)] | 0.26 |

Data expressed as mean percent change with 95% confidence interval. BMI, Body mass index; HOMA2- IR, Homeostatic model assessment method-insulin resistance; NEFA, Non-esterified fatty acids; Adipo- IR, adipose tissue-insulin resistance; Hb1Ac; glycated haemoglobin; Apo A-I, Apolipoprotein A-I; Apo B, Apolipoprotein B; HDL-C, High density lipoprotein-cholesterol; LDL-C, Low density lipoprotein- cholesterol; VLDL-C, Very low density lipoprotein-cholesterol; TG, Triglycerides. CEC, Cholesterol efflux capacity; MCP-1, Monocyte chemoattractant protein-1; IL-10, Interleukin 10; TNF-alpha, Tumor necrosis factor-alpha.

**Table S3: Comparison of the baseline characteristics of individuals with obesity and diabetes and individuals with obesity and non-diabetes**

| **Variable** | **Obese with Diabetes (n = 22)** | **Obese and Non-diabetes (n = 34)** | **p - value** |
| --- | --- | --- | --- |
| BMI (kg/m2) | 46.0 ± 6.0 | 45.9 ± 8.1 | 0.9 |
| Glucose (mg/dl) | 131.7 ± 35.5 | 101.5 ± 23.4 | 0.004 |
| Insulin (mIU/l) | 19.2 (9.5 – 24.0) | 15.7 (13.6 – 23.8) | 0.65 |
| HOMA2-IR | 3.2 ± 2.0 | 3.1 ± 2.4 | 0.77 |
| NEFA (mmol/l) | 0.8 ± 0.36 | 0.8 ± 0.43 | 0.95 |
| ADIPO-IR | 77.2 (48.0 – 125.0) | 82.5 (52.0 – 164.5) | 0.78 |
| Apo A-I (mg/dl) | 106.0 ± 13.8 | 108.8 ± 25.0 | 0.63 |
| Apo B (mg/dl) | 80.6 ± 20.8 | 84.7 ± 17.8 | 0.43 |
| Hb1Ac | 7.7 ± 1.9 | 5.9 ± 0.7 | 0.0001 |
| TC (mg/dl) | 175.3 ± 71.4 | 170.2 ± 24.5 | 0.70 |
| HDL-C (mg/dl) | 40.4 ± 9.8 | 43.5 ± 9.6 | 0.22 |
| LDL-C (mg/dl) | 99.2 ± 27.0 | 103.0 ± 24.7 | 0.57 |
| VLDL-C (mg/dl) | 24.8 ± 9.2 | 25.2± 9.1 | 0.90 |
| TG (mg/dl) | 143.1± 44.1 | 141.9 ± 45.0 | 0.92 |
| CEC (A.U.) | 0.90 ± 0.14 | 0.94 ± 0.13 | 0.22 |
| Adiponectin (µg/ml) | 5.6 ± 2.6 | 5.7 ± 1.6 | 0.84 |
| Log Resistin | 2.2 ± 0.80 | 2.3 ± 0.65 | 0.8 |
| Log MCP-1 | 1.9 ± 0.45 | 1.6 ± 0.37 | 0.04 |
| IL-10 (pg/ml) | 0.48 ± 0.55 | 0.49 ± 0.75 | 0.52 |
| TNF alpha (pg/ml) | 2.5 ± 2.17 | 1.9 ± 1.23 | 0.48 |

Data expressed as mean ± standard deviation or median with interquartile range. BMI, Body mass index; HOMA2-IR, Homeostatic model assessment method-insulin resistance; NEFA, Non-esterified fatty acids; Adipo-IR, adipose tissue-insulin resistance; Hb1Ac; glycated hemoglobin; Apo A-I, Apolipoprotein A-I; Apo B, Apolipoprotein B; HDL-C, High density lipoprotein-cholesterol; LDL-C, Low density lipoprotein-cholesterol; VLDL-C, Very low density lipoprotein-cholesterol; TG, Triglycerides. CEC, Cholesterol efflux capacity; MCP-1, Monocyte chemoattractant protein-1; IL-10, Interleukin 10; TNF- alpha, Tumor necrosis factor-alpha.

**Table S4: Comparison of the variables after surgery in individuals with obesity and diabetes and individuals with obesity and non-diabetic**

| **Variable** | **Obese with Diabetes (n = 18)** | **Obese and Non-diabetes (n**  **= 23)** | **p-value** |
| --- | --- | --- | --- |
| BMI (kg/m2) | 38.4 ± 4.9 | 37.5 ±7.3 | 0.7 |
| Glucose (mg/dl) | 103.0 ± 16.5 | 94.8 ± 13.9 | 0.11 |
| Insulin (mIU/l) | 14.3 (9 – 16.2) | 8.5 (4.7 – 10.4) | 0.005 |
| HOMA2-IR | 2.2 ± 1.8 | 1.3 ± 0.47 | 0.04 |
| NEFA (mmol/l) | 0.76 ± 0.33 | 0.7 ± 0.31 | 0.60 |
| ADIPO-IR | 60. 4 (35.7 – 118.9) | 26.1 (20.4 – 72.8) | 0.15 |
| Apo A-I (mg/dl) | 128.7 ± 17.0 | 131.0 ± 23.6 | 0.73 |
| Apo B (mg/dl) | 103.5 ± 16.8 | 97.4 ± 17.0 | 0.30 |
| Hb1Ac | 6.3 ± 0.85 | 5.4 ± 0.8 | 0.002 |
| TC (mg/dl) | 161.2 ± 26.9 | 170.1 ± 31.9 | 0.32 |
| HDL-C (mg/dl) | 37.3 ± 5.3 | 41.2 ± 6.4 | 0.042 |
| LDL-C (mg/dl) | 94.8 ± 31.5 | 113.7 ± 29.0 | 0.040 |
| VLDL-C(mg/dl) | 25.0 ± 8.1 | 20.2 ± 9.3 | 0.07 |
| TG (mg/dl) | 133.09 ± 37.9 | 105.0 ± 44.4 | 0.03 |
| CEC (A.U.) | 1.0 ± 0.18 | 1.0 ± 0.16 | 0.93 |
| Adiponectin (µg/ml) | 6.3 ± 2.7 | 7.6 ± 1.9 | 0.14 |
| Log Resistin | 2.03 ± 0.80 | 2.03 ± 0.74 | 0.98 |
| Log MCP-1 | 1.8 ± 0.31 | 1.5 ± 0.58 | 0.01 |
| IL-10 (pg/ml) | 0.26 (0.18 – 0.70) | 0.17 (0.08 – 0.27) | 0.12 |
| TNF alpha (pg/ml) | 2.4 ±1.3 | 2.6 ±1.7 | 0.68 |

Data expressed as mean ± standard deviation or median with interquartile range. BMI, Body mass index; HOMA2-IR, Homeostatic model assessment method-insulin resistance; NEFA, Non-esterified fatty acids; Adipo-IR, adipose tissue-insulin resistance; Hb1Ac; glycated hemoglobin; Apo A-I, Apolipoprotein A-I; Apo B, Apolipoprotein B; HDL-C, High density lipoprotein-cholesterol; LDL-C, Low density lipoprotein-cholesterol; VLDL-C, Very low density lipoprotein-cholesterol; TG, Triglycerides. CEC, Cholesterol efflux capacity; MCP-1, Monocyte chemoattractant protein-1; IL-10, Interleukin 10; TNF- alpha, Tumor necrosis factor-alpha.

**Table S5: Comparison of the percent change in variables after surgery between individuals with obesity and diabetes and individuals with obesity and non-diabetic**

| **Variable** | **Mean percent change [95% Conf. Interval]** | **Mean percent change [95% Conf. Interval]** | **p-value** |
| --- | --- | --- | --- |
| BMI (kg/m2) | -18.3 [(-21.1) – (15.4)] | -17.0 [(-20) – (13.6)] | 0.5 |
| Glucose (mg/dl) | -18.0 [(-27.9) – (-7.7)] | 12.0 [(-22.4) – (46.4)] | 0.05 |
| Insulin (mIU/l) | 2.5 [(-46.5) – (51.5)] | -52.2 [(-61.2) – (-43.4)] | 0.017 |
| HOMA2-IR | -6.1 [(-50.0) – (37.5)] | -48.0 [( -60.5) – (-35.7)] | 0.11 |
| NEFA (mmol/l) | 33.4 [(-29.4) – (96.3)] | 21.0 [(-36.2) – (78.2)] | 0.78 |
| ADIPO-IR | -12.0 [(-76.8) – (52.6)] | -22.0 [(-77.9) – (33.0)] | 0.81 |
| Apo A-I (mg/dl) | 24.3 [(14.7) – (33.9)] | 25.5 [(8.8) – (42.1)] | 0.90 |
| Apo B (mg/dl) | 29.3 [(13.1) – (45.5)] | 22.1 [(10.9) – (33.3)] | 0.44 |
| Hb1Ac | -18.0 [(-25.2) – (-10.4)] | -12.0 [(-17.5) – (-6.1)] | 0.20 |
| TC (mg/dl) | -3.6 [(-14.6) – (7.3)] | -1.0 [(-8.4) – (6.5)] | 0.67 |
| HDL-C (mg/dl) | -3.7 [(-14.5) – (7.0)] | -3.8 [(-13.3) – (5.7)] | 0.99 |
| LDL-C (mg/dl) | 3.6 [(-19.6) – (27.0)] | 15.6 [(-7.1) – (38.3)] | 0.45 |
| VLDL-C (mg/dl) | 10.0 [(-14.2) – (34.0)] | -7.3 [(-35.7) – (21.0)] | 0.35 |
| TG (mg/dl) | -2.3 [(-20.9) – (16.3)] | -22.0 [(-35.4) – (-8.3)] | 0.08 |
| CEC (A.U.) | 12.0 [(4.7) – (19.1)] | 11.0 [(1.9) – (20.1)] | 0.87 |
| Adiponectin (µg/ml) | 25.5 [(2.1) – (49.0)] | 41.5 [(13.8) – (69.2)] | 0.36 |
| Log Resistin | -1.0 [(-21.8) – (19.8)] | -11.2 [(-23.7) – (1.4)] | 0.40 |
| Log MCP-1 | -0.7 [(-10.5) – (8.9)] | -4.2 [(-24.3) – (15.9)] | 0.74 |
| IL-10 (pg/ml) | 222.0 [(-33.0) – (476.7)] | 740.0 [(-79.0) – (1560.0)] | 0.20 |
| TNF alpha (pg/ml) | 7.7 [(-22.7) – (38.2)] | 66.0 [(3.2) – (128.8)] | 0.05 |

Data expressed as mean percent change with 95% confidence interval. BMI, Body mass index; HOMA2- IR, Homeostatic model assessment method-insulin resistance; NEFA, Non-esterified fatty acids; Adipo- IR, adipose tissue-insulin resistance; Hb1Ac; glycated hemoglobin; Apo A-I, Apolipoprotein A-I; Apo B, Apolipoprotein B; HDL-C, High density lipoprotein-cholesterol; LDL-C, Low density lipoprotein- cholesterol; VLDL-C, Very low density lipoprotein-cholesterol; TG, Triglycerides. CEC, Cholesterol efflux capacity; MCP-1, Monocyte chemoattractant protein-1; IL-10, Interleukin 10; TNF-alpha, Tumor necrosis factor-alpha.
